# Do Children’s Attachment Security and Empathy Facilitate Story Appreciation?

**DOI:** 10.1007/s42761-025-00339-4

**Published:** 2025-12-10

**Authors:** Khatereh Borhani, Julia F. Christensen, Klaus Frieler, Zahra Aghakarimi, Ines Schindler

**Affiliations:** 1 Institute for Cognitive and Brain Sciences, Shahid Beheshti University, Tehran, Iran; 2 Department of Cognitive Neuropsychology, Max Planck Institute for Empirical Aesthetics, Frankfurt/M, Germany; 3 Max Planck Institute for Empirical Aesthetics, Frankfurt/M, Germany; 4 Seminar for Media Education, Europa-Universität Flensburg, Flensburg, Germany

**Keywords:** Aesthetic Emotions, Attachment Security, Empathy, Aesthetic Appreciation, Children’s Stories

## Abstract

In a sample of 295 native Farsi speaking children between 6 and 10 years, we investigated to what extent children experienced aesthetic emotions during story listening. Since previous research has shown that aesthetic evaluation has dispositional components, we also investigated the influence of child-reported attachment security and mother-reported child empathy on the experience of aesthetic emotions and story liking. By means of a multiple mediator model, we tested how aesthetic emotions acted as mediators of the relationship of attachment security and empathy with story liking. Results showed that attachment security was related to more pleasing and captivating emotions (with a trend for less negative emotions), and thereby, increased story liking. Empathy was linked to emotions of affinity, but these did not predict story liking, resulting in a nonsignificant effect of empathy on story liking.

**Supplementary Information:**

The online version contains supplementary material available at 10.1007/s42761-025-00339-4.

By engaging with fiction, individuals of all ages, worldwide, immerse themselves into imaginary worlds. Research with adults has shown that personality variables such as attachment and empathy are associated with absorption into and enjoyment of stories (Bálint & Bálint Kovács, 2016; Djikic et al., 2009; Hall & Bracken, 2011; Martin, 2019; Rain et al., 2017). Consumption of stories is also a common activity in children, yet research on the predictors of children’s enjoyment of stories is limited (but see Hoffner & Cantor, 1991; Jose & Brewer, 1984). Most research with children has focused on education rather than entertainment, asking what children *learn* from stories, or on children’s negative emotional responses to stories such as fear instead of on pleasure and enjoyment (Mares & Bonus, 2021). We sought to contribute to filling this research gap. Our central research question was how attachment security and trait empathy were associated with enjoying audio-recorded stories in a sample of 295 Iranian children (age 6–10), and what role emotional engagement with the stories – that is, the elicited aesthetic emotions – played as a mediator of these associations. Attachment security and empathy are related in part because of their shared link to emotional competencies (Stern & Cassidy, 2018), which suggests that both personality traits may contribute to regulating emotions in response to stories in a way that increases enjoyment and reduces potential distress.

Influences of attachment security on engagement with stories are evident during early parent–child picture book reading; securely attached toddlers are more attentive and responsive during reading interactions than less securely attached toddlers (Fletcher & Reese, 2005). Attachment-related themes such as threat, social exclusion, abandonment, and loss feature strongly in fairytales and other children’s stories (Frude & Killick, 2011). Securely attached children may be more open to explore such emotionally negative content, while it may evoke defensive responses in less securely attached children. Research with adults (Bálint & Bálint Kovács, 2016; Djikic et al., 2009; Rain et al., 2017) suggests a negative effect of attachment avoidance on transportation into story worlds and emotional engagement with stories, but a positive effect of attachment anxiety on transportation, pointing to a potential role of stories/entertainment in compensating for frustrated needs in the real world. However, this pattern of findings is qualified, for instance, by interactions between avoidance and anxiety (Rain et al., 2017), or between attachment and narrative techniques that regulate the level of proximity to a character’s mind (Bálint & Bálint Kovács, 2016).

Empathy is a multifaceted and, when it comes to enjoying stories, double-edged construct. Facets such as narrative empathy, fantasy empathy, and empathic concern have been related to character engagement (Eekhof et al., 2023) and narrative transportation (Hall & Bracken, 2011). In contrast, personal distress reduced sympathy toward characters (Eekhof et al., 2023). Studies did not find a uniform effect of empathy on enjoyment, but rather different effects depending on genre and the specific content and form of a story (Hall & Bracken, 2011; Martin, 2019). While Jose and Brewer (1984) found both direct and indirect (via suspense) positive effects of children’s character identification on story liking, we are not aware of any study testing whether trait empathy predicts story liking in children.

Emotions are not only intricately linked to attachment and empathy, but are also integral to the aesthetic experience of stories. In particular, aesthetic emotions are functionally defined as those emotions that make a direct contribution to aesthetic evaluation (Schindler et al., 2017). The Four Aesthetic Feeling States (Fafes) model (Schindler & Menninghaus, 2020) was developed to study a broad range of these emotions. It assigns diverse emotions that can be experienced in response to aesthetic materials to four factors: 1) pleasedness (e.g., joy, vitality, the feeling of beauty), 2) affinity (e.g., affection, being moved, enchantment), 3) captivation (e.g., interest, wonder, the feeling of suspense), and 4) aversion (e.g., boredom, confusion, the feeling of ugliness). While the emotions loading on each factor are distinct emotions, the factors capture their shared appraisals and action tendencies. These emotions can be assessed by means of the Aesthetic Emotions Scale (Aesthemos for adults, Schindler et al., 2017, and Aesthemos-CA for children, Schindler & Menninghaus, 2020).

## Method

The ethics committee of the Shahid Beheshti University approved the study (IR.SBU.REC.1399.071). Consent was obtained via the legal guardian of each child (more method details in the supplement).

### Participants

Three-hundred-two children (6–10-year-olds) were recruited from two kindergartens and five elementary schools in Tehran, Iran. Study advertisements were shared with parents through the schools’ WhatsApp group channels. The advertisement included a brief explanation of the experiment plus a link for signing up to the study. The percentage of children per age and gender was about equal (see Table 1).

**Table 1:** Participant Characteristics: Frequency of Ages and Gender

Age (years)	Age frequency	Gender frequency (Boy)
6	19.3%	49.1%
7	19.7%	51.7%
8	19.7%	48.3%
9	20.7%	49.2%
10	20.7%	50.8%

Exclusion criteria were: psychiatric or neurological disorders of the child (parents’ self-report), incomplete responses to Aesthemos-CA, or to the other questionnaires. Data from seven participants were incomplete and therefore excluded; final N = 295 (*M*_age_ = 8.04, *SD* = 1.41, 148 girls). Children in the age range 6–7 were attending kindergarten; children in the age range 7–8, 8–9, and 9–10 were attending first to third grade in elementary school respectively. See the supplementary material for a post-hoc power analysis, given our sample size. 

Parental education levels were also assessed. Among mothers, 16.6% had a diploma (equivalent to ‘high school diploma’ in the US – includes 11 years of education), or lower, 10.2% held an associate degree, 46.4% held a bachelor's degree, 20.3% had a master's degree, and 6.4% had a Ph.D. or equivalent doctoral degree. Among fathers, 24.4% had a diploma or lower, 11.2% held an associate degree, 32.9% had a bachelor's degree, 24.4% held a master's degree, and 7.1% had a Ph.D. or doctoral degree.

### Materials and Measures

#### Stories

We selected 14 child-suitable, audio-recorded fairytales and children’s stories (mean length = 10 min). The stories were either publicly published on the internet and national radio channels, or were purchased through audio book libraries. The stories were classified in terms of the main emotions triggered in the story, the length of the stories, and the target age group. To classify the stories according to emotions triggered in each story, two of the authors listened to all 40 stories separately and determined the main emotions. We excluded stories that did not incorporate enough emotional content, or were not suitable for 6 to 10-year-olds, or were longer than 16 min. Twenty-eight stories were retained after an initial analysis, and rated by 20 mothers in terms of familiarity of the story for their child, likability of the story by their child, the mothers’ interest in reading that story to their child, and the emotions triggered by the story. This was to ensure that the stories were equivalent in these measures. Subsequently, 14 stories were selected based on the length of the stories (outliers were removed: stories that were more than 30 min long). More details about the stories are presented in Table S2 in the supplementary material.

#### Farsi Aesthetic Emotions Scale for Children and Adolsecents (F-Aesthemos-CA)

**Fig. 1:**
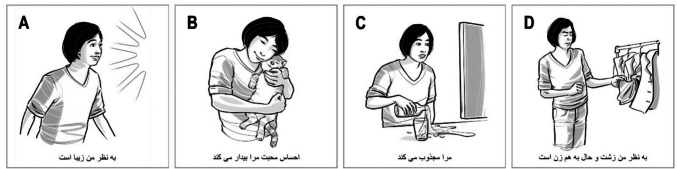
Sample Items of the F-Aesthemos-CA for Each Fafes Factor. *Note*. (**A**) The feeling of beauty (pleasedness). (**B**) Affection (affinity). (**C**) Fascination (captivation). (**D**) The feeling of ugliness (aversion)

The items of the Aesthemos-CA (Schindler & Menninghaus, 2020) consist of a drawn character displaying an emotion and a brief text labeling the emotion. Items were translated and adapted to Farsi (back-translation method; item examples in Fig. 1).

Children rated to what extent (4-point scale; “not at all”– “very”) they felt like the character “Tima” when listening to the story; they could also select the response “I do not know” (chosen for 7.2% of the ratings).

**Fig. 2:**
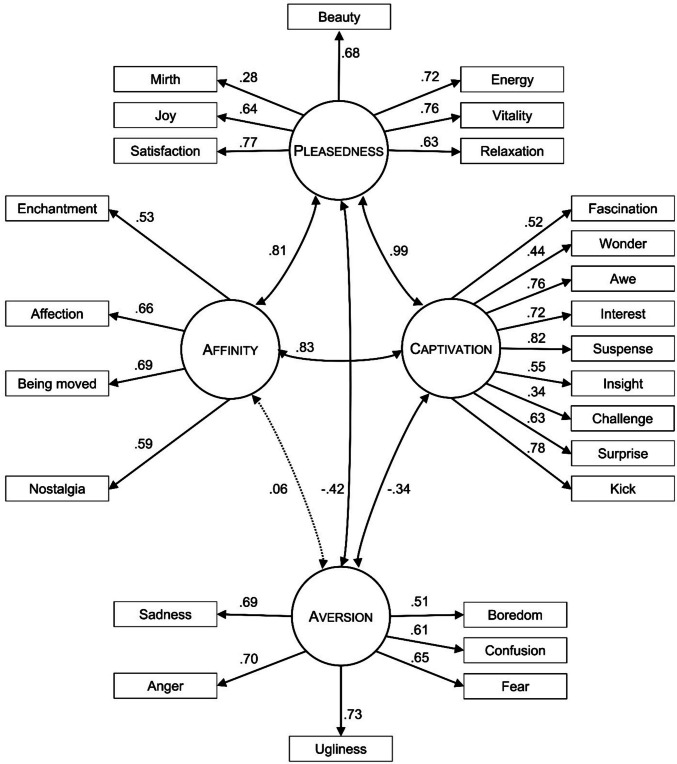
Four-Factor Model Representing the Four Aesthetic Feeling States (Fafes)

*Note*. Factor variances were fixed at 1 for model identification, which means that unstandardized and standardized parameter estimates are the same. All factor loadings and covariances shown as solid lines are significant at *p* <.001, one nonsignificant covariance is shown as dotted line. Beauty = feeling of beauty. Challenge = cognitive challenge. Kick = feeling of kick. Ugliness = feeling of ugliness. As we expected some emotion terms to be unfamiliar to children, we measured mirth with “I find it funny,” nostalgia with “makes me long for the past,” awe with “I find it overwhelming,” and the feeling of ugliness with “I find it awful.”

We ran a confirmatory factor analysis (CFA) assuming categorical data to test whether the four-factor structure (Fafes) of the original Aesthemos-CA replicated with the 26 items of the F-Aesthemos-CA. The model showed a good fit to the data, *RMSEA* =.03, 90% CI [.02,.04], *CFI* =.95, *TLI* =.95, *SRMR* =.09, and all factor loadings were greater than.20 and significant at *p* <.001 (Fig. 2). However, the pleasedness factor and the captivation factor were not cleanly separable in this data set. While a two-factor model (attraction and aversion) was superior to a one-factor model, χ^2^ (1) = 59.95, *p* <.001, and a three-factor model (pleasedness + captivation, affinity, and aversion) was superior to a two-factor model, χ^2^ (2) = 67.80, *p* <.001, adding the fourth factor (separating pleasedness and captivation) did not lead to a better model fit when compared with the three-factor model, χ^2^ (3) = 3.66, *p* =.300. As the Fafes were derived from prior empirical findings as well as theoretical considerations, we decided to stick with four factors to ensure comparability and interpretability of the findings.

For our analyses, we computed four scales to represent pleasedness (7 items), affinity (4 items), captivation (9 items), and aversion (6 items) by averaging across the items assigned to each of the scales (see Fig. 2). Descriptives and correlations of the scales are reported in Table 2.

**Table 2:** Descriptives and Correlations of Study Variable*s*

Variable	*M*	*SD*	ICC	1	2	3	4	5	6	7	8
1 Age	8.04	1.41	.00								
2 Gender (boy)	0.50	0.50	.10*	.00							
3 Attachment security (self-report)	3.21	0.50	.00	-.12**	-.03						
4 Empathy (mother’s report)	3.40	0.50	.00	.00	-.16**	.13*					
*FAFES mean scores*											
5 Pleasedness	2.77	0.74	.07*	-.19***	-.16 +	.20***	.13*				
6 Affinity	2.20	0.91	.01	.00	-.17*	.09	.13*	.47***			
7 Captivation	2.69	0.74	.02	-.10*	-.26**	.18***	.14*	.71***	.55***		
8 Aversion	1.31	0.46	.05	.14 +	-.05	-.09 +	.06	-.23***	.10***	-.14*	
9 Story liking	4.29	1.18	.02	-.23***	-.05	.01	-.06	.42***	.18***	.34***	-.19***

ICC = intraclass correlation

*** *p* <.001. ** *p* <.01. * *p* <.05. + *p* <.10

Internal consistency of the scales was estimated with McDonald’s omega (ω) and was good to acceptable: pleasedness ω =.76, 95% CI [.72,.80]; affinity ω =.64, 95% CI [.52,.73]; captivation ω =.78, 95% CI [.73,.82]; aversion ω =.61, 95% CI [.47,.70]. Further details about the F-Aesthemos-CA and the interpretation of the ICCs can be found in the supplement*.*

#### Parents’ Reports of Children’s Sympathy/Empathy

Mothers responded to five items (translated to Farsi by the first and forth authors with the back-translation method) measuring their child’s trait empathy (Zhou et al., 2019). They selected the one out of two statements that better described their child and rated whether this statement was “really true” or “sort of true” (resulting in a 4-point scale). Higher scale scores (item average; ω =.64) indicate greater empathy.

#### Farsi Kerns’ Security Scale (F-KSS)

Children’s perception of their attachment to their mothers was assessed using the 13-item Farsi version of the KSS (Verma & Talebi, 2007). For each item, participants were asked to decide which of two children is more like them, and to rate whether they are “really like” or “sort of like” that child (resulting in a 4-point scale). Higher scores on the F-KSS (average score across items; ω =.76) indicate greater attachment security.

#### Story Liking

Story liking was assessed by asking children to select how much they liked each story from five options ranging from 1 (did not like it at all) to 5 (liked it very much). This question was asked after children listened to the story and completed the F-AESTHEMOS-CA.

### Procedure

The study took place online (August 2021-February 2022). Prior to the story-listening session, mothers completed online versions of the empathy and demographic questionnaires. The children filled out the F-KSS together with the experimenter who read the items and inserted each child’s response into the online scale.

Mothers pre-selected a story for an online group reading session (different stories were offered at different dates. Mothers choose the stories based on story and available dates). After story-presentation via audio file, children responded individually to an online version of the F-Aesthemos-CA. For the 6–7-year-olds, the experimenter asked each question and noted the child’s answers. Older children filled out the scale by themselves. Further details about the procedure can be found in the supplement.

## Results

**Fig. 3:**
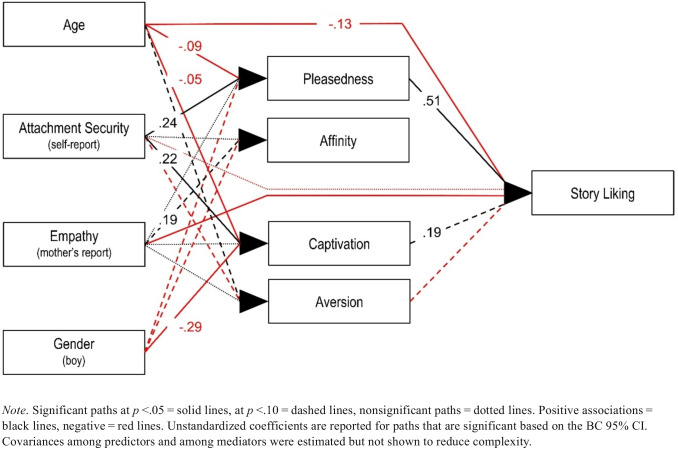
Multiple Mediator Model

A multiple mediator model (Fig. 3) was run in M*plus* to test our predictions. Given the limited number of stories in our study and the small ICCs (Table 2), it was neither feasible nor required to run a multilevel mediator model to determine indirect effects on the within-story and between-story levels. Nevertheless, we employed the *COMPLEX* option in M*plus* to address any remaining dependencies of observations within stories. In addition to computing direct and indirect effects, findings were corroborated by obtaining bias-corrected bootstrap confidence intervals (BC 95% CIs) of the effects based on 5,000 bootstrap samples. The bias-corrected bootstrap has been recommended as the best method to establish indirect effects (Preacher & Hayes, 2008; Williams & MacKinnon, 2008).

Child-reported attachment security was positively correlated with pleasedness and captivation (Table 2), and these associations were robust after controlling for age, gender, and empathy (Fig. 3; Table S3). The positive correlations of mother-reported empathy with pleasedness, affinity, and captivation (Table 2) became nonsiginificant when including rival predictors. Note that the significance level based on the *p*-value and significance based on the BC 95% CI sometimes did not match. The effect of empathy on affinity, *B* = .19, *p* =.068, BC 95% CI [> .00;.40], was significant based on the BC 95% CI, suggesting that empathy was linked to affinity. Older children and boys tended to report less positive emotional responses to the stories and age showed a direct negative association with story liking even after considering the mediated effects via the aesthetic emotion scales.

The path model sheds more light on the nonsignificant correlations of attachment security and empathy with story liking (Table 2). We found a significant indirect effect of attachment security via pleasedness on story liking, *B* = .12, *p* =.015, BC 95% CI [.05;.25], and a significant total indirect effect of attachment security via three aesthetic emotion scales, *B* = 0.18, *p* <.001, BC 95% CI [.09;.29] (excluding affinity, which was unrelated to story liking after controlling for the other three scales) on story liking. The indirect effects of attachment security via captivation, *B* = 0.04, *p* =.132, BC 95% CI [.01;.13], and via aversion, *B* = 0.02, *p* =.200, BC 95% CI [> .00;.06], on story liking were significant based on the BC 95% CI. Taken together, attachment security led to more positive (and a trend towards less negative) aesthetic emotions in response to a story and thereby increased story liking. However, this positive indirect effect was counteracted by a remaining direct negative effect of attachment security on story liking, *B* = −.21, *p* =.138, BC 95% CI [−.52;.05] (Tab. S4). Although this effect did not reach significance, it contributed to a nonsignificant total effect of attachment security on story liking.

We also found a remaining direct negative effect of empathy on story liking, *B* = −.24, *p* =.041, BC 95% CI [−.46;.01] (not significant based on the BC 95% CI). This effect was again counteracted by the overall positive, albeit nonsignificant, indirect effects of empathy on story liking, leading to a nonsignificant total effect.

## Discussion

The F-Aesthemos-CA was utilized to assess the emotional responses to stories of 295 6–10-year-old Iranian children. Children with a more secure attachment experienced positive aesthetic emotions more intensely, leading to increased story liking. This effect was primarily due to increased pleasedness as a mediator, which was the best predictor of story liking. This finding is in contrast to findings with German children, where the captivation factor was most predictive of story liking (Schindler & Menninghaus, 2020), and also findings showing that US-American children can enjoy suspense independent of the happy ending of a story (Hoffner & Cantor, 1991; Jose & Brewer, 1984). Together with the finding of pleasedness and captivation being hard to separate in our sample of Iranian children, this raises interesting future cross-cultural research questions regarding the extent to which story liking depends on pleasurable emotions. The high intercorrelation of pleasedness and captivation in the Farsi version could possibly be due to the fact that the meanings and usages of the pleasedness and captivation items differ in Western and Eastern cultures. Some items like “suspenseful” (belonging to the captivation factor) had to be translated into Farsi in a more general way since the exact meaning of this word in Farsi was deemed to be hard to comprehend by children and is not usually used by non-experts in art to describe feelings. Other items from the captivation factor, namely “fascinates me” and “sparks my interest”, were translated into Farsi in a way that would be comprehended as unequivocally positive. Therefore, it is possible that in the F-Aesthemos-CA, the captivation factor contains more positive items than the German version. The fact that the pleasedness and captivation factors of the Fafes model were not clearly separable in our sample of Iranian children also raises the conceptual question of whether Berlyne’s (1963) distinction between pleasure and interest, which is captured by these two factors, might not be as clear in Non-Western cultures. While Germans and US-Americans can easily conceive of stories that are unpleasant yet enjoyed for the feelings of suspense and fascination that they elicit, a positive aesthetic evaluation in the absence of pleasing emotions might seem strange in other cultures (and not just for children). It will be important to test whether our findings replicate in future research. Is pleasedness the best predictor of story liking for Iranian children (and children from other Non-Western cultures), while captivation is the best predictor in Western cultures? Is this finding limited to children or might it extend to older age groups as well?

There further was tentative evidence for attachment insecurity leading to more aversive emotions, thereby reducing story liking. Djikic et al. (2009) reported greater emotion change after reading a short story in adults higher on attachment avoidance. Attachment insecurity may lead to more intense negative emotional responses especially to stories featuring attachment-relevant themes. Greater engagement with story characters and transportation into stories, which has been linked to attachment insecurity (Rain et al., 2017), might lead to personal distress from difficulties in distinguishing own emotions from those of the story characters.

We also observed a trend for a negative effect of empathy on story liking and a positive link between empathy and the affinity factor, supporting the notion that empathy is a double-edged construct for story enjoyment. While it may enhance liking by helping relate to story chacters and story worlds, it may reduce liking by contributing to personal distress when characters experience negative events. Empathy has been linked to lesser enjoyment of suffering and graphic horror, but more enjoyment of danger, excitement, and happy endings (Martin, 2019). As previous research findings for adults were different for attachment avoidance and anxiety (Bálint & Bálint Kovács, 2016; Djikic et al., 2009; Rain & Mar, 2021; Silver & Slater, 2019) and for different dimensions of empathy (Eekhof et al., 2023; Hall & Bracken, 2011; Hoffner, 2009; Hoffner & Levine, 2005; Martin, 2019), future research would benefit from employing more detailed measures of different dimensions of attachment and empathy. Due to time constraints, we had to rely on short measures, and one measure was a child self-report (attachment), while the other was a parent report (empathy). While this was not ideal, this procedure has led to promising initial findings supporting a role of attachment security (and probably empathy) in influencing children’s enjoyment of stories.

In conclusion, our main finding is that attachment security led to more pleasing emotions in response to a story, and thereby, increased story liking. A post-hoc power simulation (see supplement) showed that power to detect these effects was good in our sample. Empathy was linked to the affinity factor, but this factor was not predictive of story liking in the mediator model. However, our power simulation showed that tests of paths involving empathy were underpowered and, therefore, our empathy findings remain inconclusive. Our simulations showed that a sample of more than 700 children would be required to corroborate the small effect of empathy on affinity suggested by the present findings with power over 80%.

As there is some evidence that the effects of attachment security and empathy depend on the content and formal elements of a story (e.g., Bálint & Bálint Kovács, 2016, Martin, 2019), it will be important to more closely investigate the interplay between a child’s personality, narrative techniques, and story content in future research rather than trying to recruit such large samples of children to test for potential general (small) effects of empathy. For instance, characters in contemporary children’s literature may be more relatable than fairytale characters and thus invite more empathy and engagement. As our study included only 14 stories (fairytales and other children’s stories), we could not test for effects of attachment and empathy depending on story type and content.

## Supplementary Information

Below is the link to the electronic supplementary material.

ESM 1 (DOCX 52.9 KB)

## Additional Information

### Data Availability

We have two OSF projects for this manuscript. The Mplus files can be found with these anonymized links: https://osf.io/m4nuj/?view_only = 9fdab36cdc5140afac453cafe87534b3 and the data files, here: https://osf.io/6kr97/?view_only = fae8a8a23aa74648aff0ddf6eeb2478f.
