# Supplementary material for: Do Children’s Attachment Security and Empathy Facilitate Story Appreciation?
Source: Affect Sci. 2025 Dec 10;6(4):647–55. doi: 10.1007/s42761-025-00339-4 (PMC12894583; doi:10.1007/s42761-025-00339-4)
Supplement: Supplementary file 1 — (DOCX 52.9 KB) [file 42761_2025_339_MOESM1_ESM.docx]

**SUPPLEMENTARY MATERIAL FOR:**

**Do Children’s Attachment Security and Empathy Facilitate Story Appreciation?**

This document contains additional information on methods and results, additional analyses, additional considerations for the discussion, Tables **S1** to **S3**.

**Method (Additional Information)**

**Determination of Sample Size**

This study was conducted for a master’s thesis. As there were no prior studies that allowed us to determine expected effect sizes for our central model parameters, we did not run power calculations prior to the study. We rather recruited as many children as possible within the available timeframe for running the study.

After running our analyses, we conducted a Monte Carlo study in M*plus* to calculate power given our sample size and parameter estimates. A population model was specified based on the obtained parameter estimates and assuming 21 children who listened to one out of 14 stories, resulting in a sample size of 294. The statistical model was estimated for each of 5,000 randomly generated data sets drawn from this population. We ran a multilevel model and specified our mediator model only on the within level (note that the “COMPLEX” option is not available for Monte Carlo studies, but a model specified only on the within level yields that exact same results).

We applied the following criteria to evaluate the results (see Muthén & Muthén, 2002): Both parameter bias and standard error bias should be below 5%, with values below 10% considered acceptable for standard error bias. Coverage, which is the percentage of generated data sets for which the specified population value of a model parameter lies within the estimated confidence interval for this parameter, should be close to 0.95 and remain between 0.91 and 0.98. Power above 0.80 was considered good, power of 0.70 as the lowest acceptable value.

As can be seen in Table S1, our criteria for bias and coverage were met. However, power was below 0.70 for several model paths. We found power above 0.70 for the significant paths connecting attachment security with pleasedness and captivation and for the path from pleasedness to story liking. Power to reveal our central finding that pleasedness mediates the association of self-reported attachment security with story liking was thus good. In contrast, the findings regarding the role of mother-reported empathy remain inconclusive, as power to detect small associations of empathy with the Fafes factors was too low. We ran additional simulations to determine how many children per story would be required to achieve power above 0.80 for the path from empathy to affinity and found that this would be 53 children per story, resulting in a sample size of 742 (power = 0.807).

**Table S1**

*Results of Monte Carlo Study for Post-Hoc Power Estimation (N = 294, 14 Stories, 21 Children per Story, 5,000 Replications)*

|  | **Parameter estimate** | | | **Standard error** | | | **95% Coverage** | **% Sig. coefficient (power)** |
| --- | --- | --- | --- | --- | --- | --- | --- | --- |
| **Path** | **Population** | **Average** | **Bias** | **Population** | **Average** | **Bias** |  |  |
| **Age → Pleasedness** | -0.089 | -0.0889 | -.0011 | 0.0262 | 0.0245 | -.0649 | 0.915 | **0.928** |
| **Age → Captivation** | -0.048 | -0.0481 | .0021 | 0.0249 | 0.0233 | -.0643 | 0.913 | 0.539 |
| Age → Aversion | 0.040 | 0.0397 | -.0075 | 0.0193 | 0.0178 | -.0777 | 0.916 | 0.592 |
| **Attachment → Pleasedness** | 0.244 | 0.2437 | -.0012 | 0.0827 | 0.0785 | -.0508 | 0.916 | **0.844** |
| Attachment → Affinity | 0.134 | 0.1333 | -.0052 | 0.1053 | 0.0984 | -.0655 | 0.918 | 0.310 |
| **Attachment → Captivation** | 0.223 | 0.2226 | -.0018 | 0.0823 | 0.0786 | -.0450 | 0.919 | **0.785** |
| Attachment → Aversion | -0.079 | -0.0784 | -.0076 | 0.0552 | 0.0512 | -.0725 | 0.912 | 0.357 |
| Empathy → Pleasedness | 0.133 | 0.1319 | -.0083 | 0.0853 | 0.0792 | -.0715 | 0.911 | 0.399 |
| Empathy → Affinity | 0.186 | 0.1854 | -.0032 | 0.1065 | 0.0999 | -.0620 | 0.912 | 0.464 |
| Empathy → Captivation | 0.143 | 0.1421 | -.0063 | 0.0856 | 0.0794 | -.0724 | 0.908 | 0.437 |
| Empathy → Aversion | 0.068 | 0.0669 | -.0162 | 0.0547 | 0.0510 | -.0676 | 0.913 | 0.287 |
| Gender → Pleasedness | -0.178 | -0.1793 | .0073 | 0.0824 | 0.0763 | -.0740 | 0.913 | 0.634 |
| Gender → Affinity | -0.219 | -0.2185 | -.0023 | 0.1060 | 0.0984 | -.0717 | 0.918 | 0.587 |
| **Gender → Captivation** | -0.289 | -0.2895 | .0017 | 0.0834 | 0.0774 | -.0719 | 0.910 | **0.938** |
| **Pleasedness → Story liking** | 0.509 | 0.5096 | .0012 | 0.1222 | 0.1137 | -.0696 | 0.912 | **0.983** |
| Captivation → Story liking | 0.187 | 0.1862 | -.0043 | 0.1169 | 0.1115 | -.0462 | 0.919 | 0.405 |
| Aversion → Story liking | -0.218 | -0.2196 | .0073 | 0.1383 | 0.1273 | -.0795 | 0.913 | 0.417 |
| **Age → Story liking** | -0.133 | -0.1336 | .0045 | 0.0443 | 0.0413 | -.0677 | 0.913 | **0.864** |
| Attachment → Story liking | -0.213 | -0.2129 | -.0005 | 0.1250 | 0.1174 | -.0608 | 0.916 | 0.460 |
| **Empathy → Story liking** | -0.242 | -0.2430 | .0041 | 0.1266 | 0.1167 | -.0782 | 0.912 | 0.550 |

**Note**. Bias = (average estimate – population value)/population value. Power scores > 0.700 are highlighted in bold.

**Materials and Measures**

**Table S2**

*The List of Stories in This Study*

| Story | Short description | Author | *n* participants by age (years) | | | | |
| --- | --- | --- | --- | --- | --- | --- | --- |
|  |  |  | 6 | 7 | 8 | 9 | 10 |
| The pumpkin | An elderly woman outsmarts wild animals on her journey to visit her daughter by convincing them to wait and eat her when she returns plump but she escapes their plan by hiding in a pumpkin and makes it safely home. | Parviz Kalantary | 7 | 2 | 3 | 4 | 5 |
| The horse and the snail | A race was held between a horse and a snail, which had an interesting result. | Unknown (Andrew Smith) | 4 | 4 | 4 | 4 | 8 |
| The little match girl | A little girl trying to sell matches in the cold winter night. Failing to sell the matches, she lights them up and in the warmth of the matches she sees her grandmother. | Hans Christian Andersen | 7 | 1 | 2 | 8 | 5 |
| The lying shepherd (also known as The boy who cried wolf) | A shepherd boy who continuously deceives villagers by pretending that a wolf is attacking the flock in his town. | Aesop | 2 | 5 | 4 | 4 | 4 |
| Uninvited guests | A hospitable old woman who warmly welcomes any animal that comes into her house. | Farideh Farjam | 2 | 7 | 4 | 6 | 4 |
| The ugly duckling | A duckling that, upon hatching alongside its siblings, is ridiculed and excluded due to being considered unattractive. | Hans Christian Andersen | 3 | 5 | 2 | 4 | 4 |
| The Parrot and the grocer | A grocer who had a charming green parrot that entertained customers by singing and speaking kindly, thus boosting the grocer’s sales. | Rumi | 4 | 4 | 3 | 6 | 4 |
| Hassan, the bald | Hasan, known as the bald boy due to his hairlessness, is lazily eating and sleeping all day, prompting his mother to kick him out and force him to find a job. | Sahar Azimi | 2 | 7 | 8 | 2 | 4 |
| Arash the archer | During the reign of King Manouchehr of Iran and King Afrasiab of Turan, a territorial dispute led to the selection of Arash the archer as the Iranian archer to determine the border between the two warring nations after the Iran-Turan war. | Rosa Azarvand | 2 | 6 | 4 | 4 | 5 |
| A bear called Paddington | The story follows a young bear who travels from Darkest Peru to London and is welcomed by the Brown family, marking the start of their adventures together. | Michael Bond | 4 | 3 | 8 | 2 | 1 |
| Snow-white and Rose-red | Two sisters, Snow-White and Rose-Red, live with their mother in a small cottage by the woods, with Snow-White being quiet and indoorsy while Rose-Red is lively and outdoorsy. | Brothers Grimm | 5 | 3 | 6 | 3 | 2 |
| King Thrushbeard | Many years ago, a great king had a beautiful daughter and was looking for a suitable husband for her. Although the princess was very beautiful, she was also very proud and rude and misbehaved with all the princes who proposed her. | Hans Christian Andersen | 5 | 3 | 5 | 3 | 6 |
| Thumbling | Thumbling is a beautiful little girl the size of a knuckle, whom her mother wished to have, and who came out of a flower leaf through the magic of a witch. | Bobby Norfolk | 5 | 4 | 4 | 4 | 4 |
| The tug of war | Tortoise seeks friendship but proves his intelligence by challenging Elephant and Hippo to a tug of war. | John Burningham | 5 | 4 | 2 | 5 | 5 |

***Further Information on the F-Aesthemos-CA***

The current study is the first attempt to adapt and translate the Aesthemos-CA scale to a non-Western culture, Iran. We expected that, as in the German version, the Farsi version of the Aesthemos-CA would show good structural and internal validity in an Iranian child sample.

The 28 items of the original Aesthetic Emotions Scale for Children and Adolescents (Aesthemos-CA; Schindler, *in preparation*; Schindler & Menninghaus, 2020) consist of the drawn character “Kim” displaying a target emotion and a German text underneath the drawing stating in the first-person how the character feels. For the Farsi version, the character was slightly modified by the professional drawer who produced the original drawings to look like a Middle Eastern character: darker color of hairs and eyes, wider nasal septum, and rounder face oval (see examples in Figure 1 in the paper). The character was designed to have no specific gender and was given the unisex name “Tima” for the Farsi version of the questionnaire.

Two out of 28 items were dropped from all analyses. The item “I find it moving” was not used. As we anticipated the emotion “being moved” to be difficult to assess, we tried measuring it with two different items and found that the second item “touches me” was better understood by the children. The item “wows me” was dropped because the emotion “enthusiasm” is no longer part of the original Aesthemos-CA.

As each child listened to one out of 14 stories, the 26 ratings of emotional responses to stories are clustered within stories. We computed intraclass correlations (ICCs; reported in Table 2 in the paper) to determine how much variance in the items is accounted for by the cluster variable story. The highest ICCs were obtained for sadness, ICC = .17, *p* = .062, and joy, ICC = .12, *p* = .015, the lowest for confusion, ICC = .00, *p* = .958. ICCs were significantly different from 0 at *p* < .05 for joy, mirth, and suspense and nonsignificant for the remaining 23 items. The low and mostly nonsignificant ICCs show that the variance in emotion ratings is attributable to children’s individual response to the stories rather than to the stories themselves. Nevertheless, we used the *COMPLEX* option in M*plus* Version 8.4 (Muthén & Muthén, 1998-2017) in all analyses presented in the paper to account for any remaining nonindependence of observations within stories and to obtain corrections to the standard errors and chi-square test of model fit. The data of all 295 children were employed in all analyses, as M*plus* can handle cases with missing data.

**Procedure**

The study took place online on Skyroom or Whereby during the Covid-19 pandemic (August 2021 – February 2022). Families who gave their consent for their child to participate in this study were invited to join an online story listening group session. Prior to the session, a list of all stories (see Table S2) was sent to the parents. They selected which story they would prefer their child to listen to. During the sessions, the experimenter introduced herself, explained the procedure and asked the child to briefly introduce themselves. Then, the auditory file of the story was played for the children. Subsequently, individual online sessions were held for each child and the online F-Aesthemos-CA was applied. For the 6–7-year-old children, the experimenter asked each question and wrote down the child’s answers. Children older than 7 years were able to read and fill out the scale by themselves. The experimenter held the session according to a pre-designed protocol. Prior to completing the Aesthemos-CA session, on a separate day, mothers filled out online versions of the empathy and demographic questionnaires and children were asked to fill out the attachment scale while the experimenter was reading the items and inserting the child’s response into the online scale. All online scales and questionnaires were designed through Porsline, an Iranian online survey service. Children and their parents were thanked in the end without receiving any incentives.

**Results (Additional Information)**

A multiple mediator model was specified to test our predictions on aesthetic emotions as mediators of the relationship of attachment security and empathy with story liking. We initially ran a mediator model including all possible paths between the predictor variables attachment security, empathy, age, and gender (the latter two were included as control variables), the Fafes scales as mediator variables, and the outcome story liking. As the paths from age to affinity, from gender to aversion, from gender to story liking, and from affinity to story liking were nonsignificant and essentially zero, we deleted these four paths from the model. Note that we did not delete nonsignificant paths that were relevant to testing our predictions. Figure 3 (in the paper) gives an overview of the final path model. The parameter estimates for the model paths as well as indirect and total effects of the predictor variables are reported in Table S3. Note that all significant indirect effects represent small effects. For instance, by way of attachment’s indirect pathways through pleasedness, captivation, and aversion, an increase in attachment security by one point on the 4-point scale would lead to an increase in story liking by 0.18, which amounts to *d* = 0.15 (based on the *SD* of story liking).

**Table S3**

*Mediation Findings: Attachment Security and Empathy Predict Story Liking through Emotional Responses to Stories*

|  | **Unstandardized results** | | | **BC 95% CI** | | **Standardized results** |
| --- | --- | --- | --- | --- | --- | --- |
| **Path** | **Estimate** | **SE** | ***p*** | **LL** | **UL** | **Estimate** |
| ***Paths from predictors to mediators*** |  |  |  |  |  |  |
| **Age → Pleasedness** | -0.09 | 0.02 | < .001 | -0.13 | -0.04 | -.17 |
| **Age → Captivation** | -0.05 | 0.02 | .042 | -0.09 | -0.00 | -.09 |
| Age → Aversion | 0.04 | 0.02 | .095 | -0.00 | 0.09 | .12 |
| **Attachment → Pleasedness** | 0.24 | 0.08 | .004 | 0.08 | 0.41 | .17 |
| Attachment → Affinity | 0.13 | 0.14 | .342 | -0.15 | 0.40 | .07 |
| **Attachment → Captivation** | 0.22 | 0.07 | .002 | 0.08 | 0.37 | .15 |
| Attachment → Aversion | -0.08 | 0.05 | .086 | -0.18 | 0.00 | -.09 |
| Empathy → Pleasedness | 0.13 | 0.09 | .116 | -0.01 | 0.32 | .09 |
| Empathy → Affinity | 0.19 | 0.10 | .068 | **0.00** | **0.40** | .10 |
| Empathy → Captivation | 0.14 | 0.11 | .184 | -0.04 | 0.38 | .10 |
| Empathy → Aversion | 0.07 | 0.05 | .146 | -0.01 | 0.17 | .07 |
| Gender → Pleasedness | -0.18 | 0.11 | .090 | -0.40 | 0.01 | -.12 |
| Gender → Affinity | -0.22 | 0.13 | .079 | -0.47 | 0.02 | -.12 |
| **Gender → Captivation** | -0.29 | 0.10 | .004 | -0.50 | -0.10 | -.20 |
| ***Paths from mediators to outcome*** |  |  |  |  |  |  |
| **Pleasedness → Story liking** | 0.51 | 0.12 | < .001 | 0.29 | 0.77 | .32 |
| Captivation → Story liking | 0.19 | 0.10 | .069 | **0.01** | **0.41** | .12 |
| Aversion → Story liking | -0.22 | 0.13 | .085 | -0.49 | 0.01 | -.09 |
| ***Remaining direct paths from predictors to outcome*** | | | | | | |
| **Age → Story liking** | -0.13 | 0.04 | .001 | -0.21 | -0.04 | -.16 |
| Attachment → Story liking | -0.21 | 0.14 | .138 | -0.52 | 0.05 | -.09 |
| **Empathy → Story liking** | -0.24 | 0.12 | .041 | **-0.46** | **0.01** | -.10 |
| ***Indirect and total effects*** |  |  |  |  |  |  |
| **Indirect effect: Attachment → Pleasedness → Story liking** | 0.12 | 0.05 | .015 | 0.05 | 0.25 | .05 |
| Indirect effect: Attachment → Captivation → Story liking | 0.04 | 0.03 | .132 | **0.01** | **0.13** | .02 |
| Indirect effect: Attachment → Aversion → Story liking | 0.02 | 0.01 | .200 | **0.00** | **0.06** | .01 |
| **Total indirect effect: Attachment → Mediators → Story liking** | 0.18 | 0.05 | < .001 | 0.09 | 0.29 | .08 |
| Total effect: Attachment → Story Liking | -0.03 | 0.13 | .810 | -0.28 | 0.21 | -.01 |
| Indirect effect: Empathy → Pleasedness → Story liking | 0.07 | 0.05 | .182 | -0.00 | 0.21 | .03 |
| Indirect effect: Empathy → Captivation → Story liking | 0.03 | 0.03 | .380 | -0.00 | 0.14 | .01 |
| Indirect effect: Empathy → Aversion → Story liking | -0.02 | 0.01 | .174 | -0.04 | 0.00 | -.01 |
| Total indirect effect: Empathy → Mediators → Story liking | 0.08 | 0.07 | .252 | -0.03 | 0.24 | .03 |
| Total effect: Empathy → Story Liking | -0.16 | 0.13 | .227 | -0.41 | 0.11 | -.07 |
| ***R^2^ for mediators and outcome*** |  |  |  |  |  |  |
| Pleasedness | .09 | .03 | .001 |  |  |  |
| Affinity | .04 | .03 | .193 |  |  |  |
| Captivation | .09 | .04 | .011 |  |  |  |
| Aversion | .03 | .02 | .236 |  |  |  |
| Story liking | .23 | .05 | < .001 |  |  |  |

*Note*. SE = standard error. BC 95% CI = bias-corrected 95% confidence interval for unstandardized estimate based on 5,000 bootstrap samples. LL = lower limit. UL = upper limit. Paths that are significant at *p* < .05 are highlighted in bold. The BC 95% CI is highlighted in bold if it indicates a significance level that differs from that of the unstandardized estimate.

**Discussion (Additional Considerations and Future Research Directions)**

**Effects of Age and Gender**

We found an effect of age on positive aesthetic emotions and on story liking: older children reported fewer positive emotional responses to stories and liked the stories less. A similar finding of more negative evaluations of stories with increasing child age was already reported by Jose and Brewer (1984), who speculated that this might be attributable to more socially desirable responding of younger children (especially as they were interviewed individually) or to the stories being more interesting to younger children. In our study as well, younger children filled out the questionnaire together with the experimenter while older children did so on their own, which might have encouraged more socially desirable responses of younger children. However, this may also be a substantive finding on the development of children’s evaluation of stories and requires careful future investigation.

Gender also influenced the extent to which children reported positive emotions. Boys exhibited less captivation. Perhaps because, compared to boys, girls tend to express more positive emotions, and notably, this contrast becomes more prominent as children age (Chaplin & Aldao, 2013).

**References**

Chaplin, T. M., & Aldao, A. (2013). Gender differences in emotion expression in children: a meta-analytic review. *Psychological Bulletin, 139*(4), 735-765. doi:10.1037/a0030737

Jose, P. E., & Brewer, W. F. (1984). Development of story liking: Character identification, suspense, and outcome resolution. *Developmental Psychology, 20*(5), 911. <https://psycnet.apa.org/record/1985-00860-001>

Muthén, L. K., & Muthén, B. O. (1998-2017). *Mplus User’s Guide* (8th ed.). Muthén & Muthén. <https://www.statmodel.com/download/usersguide/MplusUserGuideVer_8.pdf>

Muthén, L. K., & Muthén, B. O. (2002). How to use a Monte Carlo study to decide on sample size and determine power. Structural Equation Modeling, 9(4), 599–620. https://doi.org/10.1207/S15328007SEM0904_8

Schindler, I. (2024, under review). Emotional underpinnings of aesthetic experience. In I. Schindler & F. Degé (Eds.), *Development of aesthetic experience: Towards an integrative empirical approach.* Cambridge University Press. Chapter in preparation.
